# Monitoring the neural activity associated with praying in Sahaja Yoga meditation

**DOI:** 10.1186/s12868-023-00828-x

**Published:** 2023-11-13

**Authors:** Oscar Perez-Diaz, Alfonso Barrós-Loscertales, Uffe Schjoedt, José L. González-Mora, Katya Rubia, José Suero, Sergio Elías Hernández

**Affiliations:** 1 Universidad de La Laguna, Tenerife, Spain; 2 Departamento de Psicología Básica, Clínica y Psicobiología, Universitat Jaume I, Castellón, Spain; 3 Department of the Study of Religion, Aarhus University, Aarhus, Denmark; 4 Instituto Universitario de Neurociencia, Universidad de La Laguna, Tenerife, Spain; 5 Institute of Psychiatry, Psychology and Neuroscience, King’s College London, London, UK; 6 Centro de Salud Jazmín, Sermas, Madrid, Spain

**Keywords:** Prayer, Meditation, Sahaja Yoga, Social cognition, Theory of mind, Belief

## Abstract

**Background:**

Sahaja Yoga Meditation draws on many religious traditions and uses a variety of techniques including Christian prayer to reach a state known as thoughtless awareness, or mental silence. While there are many studies on the neural correlates of meditation, few studies have focused on the neural correlates of praying. Thus, the aim of our research was to study the neural activity associated with the prayer practices in Sahaja Yoga Mediation, which have not been studied before, to explore effects beyond repetitive speech or “mantra effects”. Sixteen experienced Sahaja Yoga Meditation practitioners were scanned using task based functional Magnetic Resonance Imaging while performing formalised and improvised forms of praying and their equivalent secular tasks.

**Results:**

Our results showed the deactivation of bilateral thalamus during both prayers compared to secular conditions and the activation in the medial prefrontal cortex that was reduced by religious and formalised secular speech conditions but increased during improvised secular speech; similarly, frontal regions were deactivated when comparing prayers to their secular equivalents.

**Discussion:**

These results seem to depict two important factors related with praying in Sahaja Yoga Meditation merging inner concentration and social cognition. First, the perception of the surroundings mediated by the thalamus may be decreased during these prayers probably due to the establishment of inner concentration and, second, frontal deactivation effects could be related to reduced social judgement and ‘mentalizing’, particularly in the medial prefrontal cortex. Our findings suggest that praying by Sahaja Yoga Meditation practitioners is neurophenomenologically different from the social cognitive attempt of praying within Christian praying practices.

**Supplementary Information:**

The online version contains supplementary material available at 10.1186/s12868-023-00828-x.

## Background

Meditation comprises a wide range of different practices, most of them aiming to increase awareness, consciousness, inner peace, self-realization and attention, while reducing stress and anxiety [1]. Some meditation practices have a spiritual part which could create links with religion and some religions include meditation as part of their practices. The changes associated with meditation in the brain have boosted the interest of the neuroscientific community on this topic. Different reviews have reported differences in brain structure and function associated with the practice of meditation in cross-sectional and/or longitudinal studies [2, 3]. Sahaja Yoga Meditation (SYM) teaches its practitioners to reach a state known as thoughtless awareness or mental silence where thoughts are considerably reduced or completely vanished, which is contemplated as the utmost goal of meditation according to the ancient text “Yoga Sutras of Patanjali” [4]. In this state of mental silence, meditators are fully aware of themselves, continuously living each present moment in a state of peace, harmony and high efficiency [5].

Religion involves diverse cognitive tasks, behaviours, praying practices and spiritual beliefs which change across cultures. In most religions, an important goal is to communicate or to relate to the divine. Religion as defined by Koenig [6] is “a system of beliefs and practices observed by a community, supported by rituals that acknowledge, worship, communicate with, or approach the Sacred, the Divine, Ultimate Truth, Reality, or nirvana”. Previous studies have illustrated that religious cognition depends on contextual and developmental practices, individual goals, and sophisticated emotional and embodied states [7]. A particular example of such practices, where religion and social cognition merge, is the act of praying, which is a form of communication between the individual and God as a culturally transmitted entity [8]. Since praying is a language-based communication behaviour, which depends on the individual’s environment and experience, its structure as a ritual has become the primary subject in religious cognitive science, more specifically the difference between formalised and improvised forms of religious expression.

Research of religious cognition has revealed fascinating insights into the cognitive processes that underlie religious beliefs and practices. Unlike other beliefs, religion relies upon a mixture of cognitive and emotional functions governed by specific brain networks [9], which may be activated depending on the experimental design used in each study [10]. In their reviews on religious cognition, Rim et al. [11] and Grafman et al. [10] report findings on cognitive systems supporting religious beliefs and spirituality, including the frontal and anterior temporal lobes [12, 13]; medial prefrontal cortex (mPFC), inferior frontal gyrus, temporoparietal junction (TPJ) and precuneus [8, 14]; dorsomedial prefrontal cortex (dmPFC) and ventrolateral prefrontal cortex (vlPFC) [10, 15, 16]; and vmPFC, striatum and nucleus accumbens [10, 17]. Overall, the findings suggest that religious experiences involve complex cognitive and neural processes known to affect cognitive control [18].

Several imaging studies on social cognition show the involvement of the lateral, ventral and medial prefrontal cortices, the precuneus as well as TPJ cortices during social cognition tasks [19–22]. Activity in lateral prefrontal as well as medial prefrontal and parietal areas during participants’ declarations of personal interactions with God was considered similar to that observed in social cognition tasks [23, 24]. In line with this, Schjoedt et al., [8] found activation in mPFC, the TPJ, precuneus and the temporopolar region when comparing an improvised prayer with a structured one (Lord’s Prayer). The mPFC, TPJ along with the temporopolar region have been considered as the foundational areas for theory-of-mind, meaning that these are the key areas facilitating the interpretation of beliefs and intentions of other people [19, 25]. It was then proposed by the authors that communication with God in a personal improvised prayer involves brain regions similar to those mediating interpersonal interaction involving social cognition [8].

SYM uses a large variety of religious practices among which praying plays an important role. It is common that SYM practitioners recite the Lord’s Prayer from Christianity, Psalm 23 or Psalm of David from Judaism, recite the holy names of Allāh from Islam or recite the Ganesha Atharva Sheersha from Hinduism [26, 27] as a practice to facilitate the state of mental silence meditation that has been described in previous papers [28]. In this sense, the meditative state in SYM, and its related brain activity, could be dependent on the praying practices used by SYM practitioners [29]. Therefore, brain activity associated with praying as a practice to get into meditation may help to understand different meditation practices and states.

The literature divides prayers into two different forms: highly formalised, prefixed and frequently rehearsed speech acts, and non-institutional prayers that are mainly improvised actions of speech acts [30–32]. This division is also reflected in SYM in which practitioners recite the Lord’s Prayer as well as praying freely to the Divine Mother. The aim of this study was therefore to explore praying in SYM beyond repetitive speech [33], known as the “mantra-effect”. We furthermore contrasted praying with similarly constructed language tasks, given the limitations of comparing praying against resting [34, 35], which as mentioned [36] is not the best control condition as does not control for inner speech related brain activation. Therefore, similarly constructed secular language tasks were included to control for language-based activations in order to isolate the effect of the prayers [29]. Thus, we used an adapted version of the study design by Schjoedt and colleagues [8, 17], to examine improvised and formalized ways of praying as preparatory practices to mental silence meditation.

The main objective of this research was therefore to determine the neural activity associated with the prayer practices within SYM to help us further characterize the basis of this meditation technique. Although SYM has been studied from different perspectives mainly focused on the brain correlates of the state of mental silence [28, 37–39], the neural activity associated with praying has not been scientifically covered yet, which is a key objective of this study and could provide some insights on this particular phenomenology of SYM. Since SYM practitioners direct their prayers to the divine in order to achieve the state of thoughtless awareness, we expected the involvement of similar areas as those observed in the previous study in Christian prayers [8]. Specifically, as in the study in Christians we expected the involvement of regions related to social cognition such as lateral and medial prefrontal regions, the temporo-parietal junction and the precuneus. However, we also expected the involvement of other areas we observed previously to be involved during SYM such as the anterior cingulate cortex, the dorsolateral prefrontal cortex (dlPFC), vlPFC, mPFC, insula, parietal cortex, and temporal cortex [28].

## Results

Volunteers reported that they were able to concentrate and properly follow all the tasks inside the scanner despite the noise and discomfort the Magnetic Resonance Imaging (MRI) scanner generates. When asked to evaluate their performance for the different tasks from 5 “very well” to 1 “very bad”, they reported on average from well to very well, with these mean scores: Lord’s Prayer (Father) 4.5 (*SD* = 0.73), Prayer to the Divine Mother (Mother) 4.63 (*SD* = 0.62), Wishes to Santa (Santa) 4.13 (*SD* = 0.72), Poem 4.38 (*SD* = 0.62), Counting backwards 4.28 (*SD* = 0.82).

### Main effects of Prayer act

**Fig. 1:**
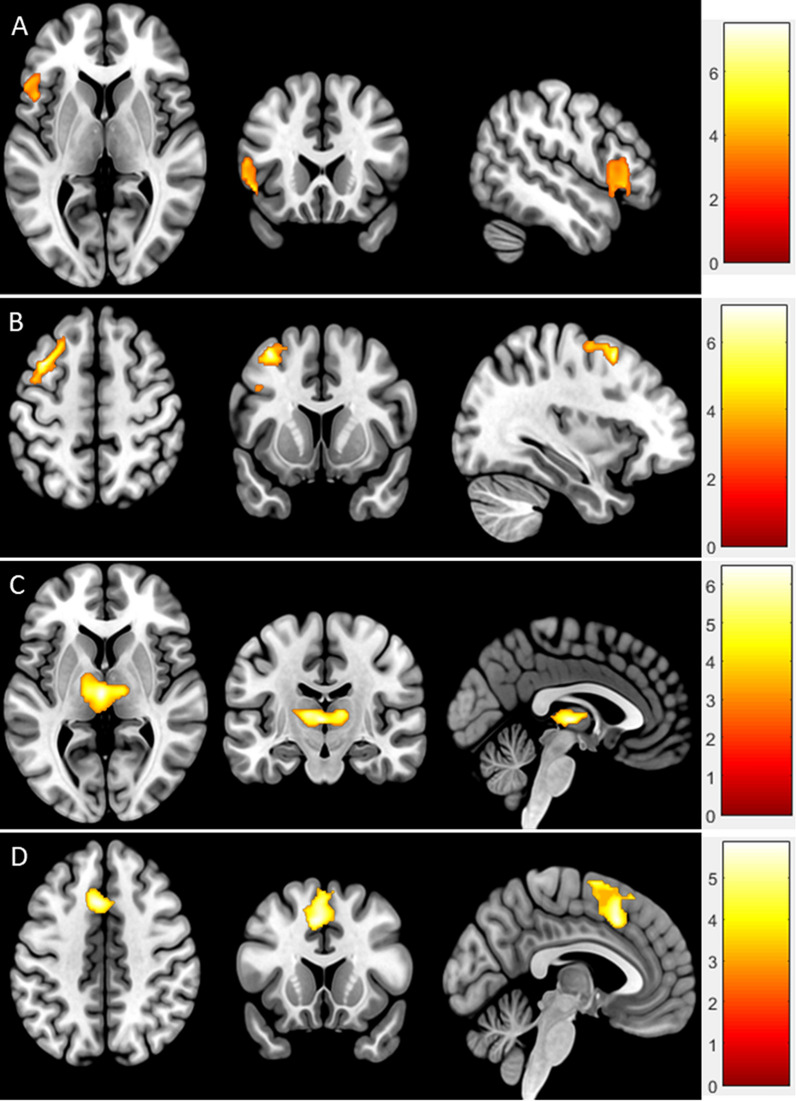
Main effects of secular domain (Santa&Poem > Mother&Father). **A** vlPFC/TP; **B** dlPFC, precentral gyrus; **C** Bilateral thalamus; **D** Supplementary motor area/ dACC. *vlPFC* ventrolateral prefrontal cortex, *TP* temporal pole, *dlPFC* dorsolateral prefrontal cortex, *dACC* dorsal anterior cingulate cortex

We compared the effects on the two prayers conditions relative to their secular contrasts (Mother&Father > Santa&Poem) in order to observe the general effects of “Domain” factor, no cluster survived this contrast. For the opposite direction, which analysed the effect of higher activation during the secular contrasts compared to the religious conditions (Santa&Poem > Mother&Father) there was a strong response in four clusters including the left vlPFC/temporal pole (TP), left dlPFC/premotor cortex, bilateral thalamus and bilateral supplementary motor area (SMA)/dorsal anterior cingulate cortex (dACC), see Table 1 and Fig. 1.

**Table 1:** Main effect of Prayer act

Cluster peak region	Side	Brodmann Area (BA)	*Z*-value	*p*-value	Cluster size (voxels)	Peak coordinate (x y z)
Main effect of religious domain (Mother & Father > Santa & Poem)						
No suprathreshold voxels						
Main effect of secular domain (Santa & Poem > Mother & Father)						
Ventrolateral prefrontal cortex/Temporal Pole	Left	45/47	4.77	0.01	365	-48 24 -6
Dorsolateral Prefrontal cortex/Precentral gyrus	Left	9/8	4.61	< 0.001	690	-34 10 54
Bilateral Thalamus	Left–Right		4.40	<0 .001	776	-2 -16 4
Supplementary Motor Area/Dorsal anterior cingulate cortex	Left	6/32	4.16	<0 .001	682	-4 16 44

### Main effect of Speech act

The effect of “Speech act” formalised versus improvised (Father&Poem > Mother&Santa) generated activity in two right hemisphere clusters: one including dlPFC and inferior frontal cortex and the other composed of inferior parietal cortex (IPC), supramarginal and angular gyrus. In the opposite direction improvised versus formalised speech (Mother&Santa > Father&Poem) showed activity in four left hemispheric clusters including Frontal eye field/Premotor cortex, and vlPFC, SMA/ACC, and dlPFC (see Table 2).

**Table 2:** Main effect of Speech act

Cluster peak region	Side	BA	*Z*-value	*p*-value	Cluster size (voxels)	Peak coordinate (x y z)
Main effect of formalised Speech act (Poem & Father > Santa & Mother)						
Dorsolateral Prefrontal Cortex/Inferior Frontal Cortex	Right	46/10	4.53	0.001	512	38 50 8
Inferior Parietal Cortex/SupraMarginal Gyrus/Angular Gyrus	Right	40	4.04	<0 .001	587	56 -42 34
Main effect of improvised Speech act (Santa & Mother > Poem & Father)						
Frontal eye Field/Pre-motor cortex	Left	6/8	5.18	0.002	450	-40 2 50
Ventrolateral Prefrontal cortex	Left	45/47	5.01	<0 .001	738	-46 26 -2
Supplementary Motor Area/dorsal anterior cingulate cortex	Left	6/32	4.77	<0 .001	726	-8 16 64
Dorsolateral prefrontal Cortex	Left	10/9	4.03	0.019	277	-24 48 28

### Interactions

**Fig. 2:**
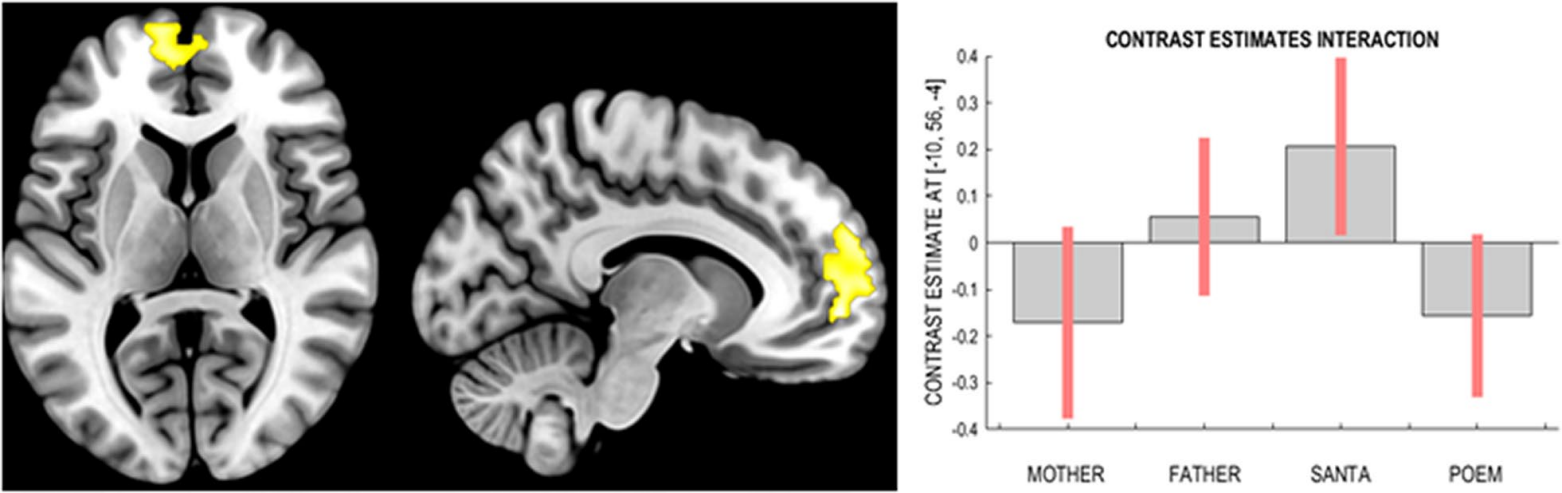
Interaction analysis results and contrast estimates

The interactions between the two factors “Domain” (religious or secular) and “Speech act” (formalised or improvised) showed activity in the left mPFC in the direction Father&Santa > Mother&Poem while no suprathreshold voxels survived in the opposite direction Mother&Poem > Father&Santa (see Table 3). This interaction shows that the “Domain” factor is affected by the “Speech act” in this region. Particularly, we observed that the activation in these regions is reduced during both religious levels and the formalised secular level but increased during the improvised secular level (see Fig. 2). This region did not overlap with any previous location of main effects analysis.

**Table 3:** Interactions

Cluster peak region	Side	BA	*Z*-value	*p*-value	Cluster size (voxels)	Peak coordinate (x y z)
Mother&Poem > Father & Santa						
No suprathreshold voxels						
Father&Santa > Mother & Poem						
Medial Prefrontal Cortex	Left–Right	10	3.87	<0 .001	740	− 10 56 − 4

### Simple contrasts

Individual contrasts comparing both types of prayers Mother vs. Father, in the direction Mother > Father showed activity in left hemisphere areas including SMA, dACC and vlPFC and TP while the opposite direction, Father > Mother, showed activity in right hemisphere areas including the temporo-parietal junction and dlPFC (see Table 4).

**Table 4:** Individual contrasts

Cluster peak region	Side	BA	*Z*-value	*p*-value	Cluster size (voxels)	Peak coordinate (x y z)
Mother > Father						
Supplementary Motor Area/Dorsal anterior cingulate cortex	Left	6/32	4.78	<0 .001	1019	-8 18 40
Ventrolateral prefrontal cortex/Temporal Pole	Left	45/44/47	4.46	0.002	417	-48 24 16
Father > Mother						
Temporo-parietal junction (Inferior Parietal Cortex, SupraMarginal Gyrus Angular Gyrus, Middle and Superior temporal Gyrus)	Right	40	4.59	< 0.001	798	56 -40 34
Dorsolateral Prefrontal Cortex	Right	46/10	4.07	<0 .001	375	38 46 0
Santa > Mother						
Superior Frontal Cortex	Left	8/9/6/10	4.33	< 0.001	1224	-10 32 50
Mother > Santa						
No suprathreshold voxels						
Poem > Father						
Supplementary Motor Area, Dorsal anterior cingulate cortex	Left–Right	6/32	4.37	< 0.001	754	-8 18 42
Premotor Cortex	Right	6/8	4.30	< 0.001	331	48 4 48
Bilateral Thalamus	Left–Right		4.08	<0 .001	939	14 -18 10
Inferior Frontal Cortex	Left	9/6	3.80	< 0.001	447	-38 0 42
Father > Poem						
No suprathreshold voxels						

The comparison between the two improvised speeches showed no activation for Mother > Santa, while the opposite condition Santa > Mother showed increased activity in the left frontal superior lobe. The comparison between the two formalised speeches showed no activation for Father > Poem, while the opposite condition Poem > Father showed an increased activity in the left frontal superior and superior middle lobe and bilateral thalamus. Poem > Father also revealed a very similar cluster to Mother > Father in the left supplementary motor area.

## Discussion

The objective of this research was to find the neural activity associated with prayer practices within SYM and also to test whether praying practices within SYM involve social brain regions as has been shown in praying within Christian practices [8]. We observed that, when compared with secular speech, praying involved a reduced activation in the thalamus in both hemispheres of the brain, and reduced activity in the left dlPFC, left vlPFC and left SMA. The same left dlPFC, left vlPFC and left SMA regions were however also decreased in activation for formalised speech versus improvised speech, possibly complying with the necessity of activating these areas for spontaneous speech production. We also observed that the activation in the medial prefrontal cortex was reduced by prayers and formalised secular speech but increased during improvised secular speech. Therefore, these results may potentially reflect the particularities of praying during SYM, where prayers are used to facilitate the later meditation state of mental silence in which practitioners perceive the state of yoga (spiritual union with God), and which may be concomitant with the observed reduced frontal activation which has not appeared in previous fMRI studies of praying in Christians [8, 17].

The results of comparing praying tasks relative to their respective secular tasks (Poem > Father, Santa > Mother or Santa&Poem > Mother&Father) always showed reduced activation in areas of the frontal lobe for both prayer tasks (Mother and Father). It is important to remark that the opposite contrasts displayed no results (Father > Poem, Mother > Santa or Mother&Father > Santa&Poem). We think that these results of deactivation at the frontal lobe during both prayers may constitute a relevant particularity of praying in SYM, especially if we consider that this was not observed with a similar paradigm in Danish Christians [8]. This deactivation of frontal areas while praying in SYM is also interesting considering that most fMRI studies made during religious experiences show greater activity in the frontal lobe [23, 40–42]. The deactivation of frontal lobe areas while praying in SYM could be related with the attitude of surrendering to God manifested by practitioners of SYM that, interestingly, seems to be more like the brain activation patterns experienced through intense Islamic Prayers, which was also correlated with decreased activity in frontal areas [43].

Similarly, the interaction analysis revealed a cluster in the mPFC, which was reduced in activation during praying (Father and Mother) and during secular formalized speech (Poem) compared to secular improvised speech (Santa). The mPFC plays a critical role in a wide range of cognitive [44] and emotional [45, 46] social processes; thus, diverse findings in this region have been reported in religious cognition and meditation studies depending on the experimental design and practices analysed [10, 47]. Particularly, the anterior mPFC has consistently appeared in social cognition and theory of mind studies [22], and has been associated with the integration of emotional and moral elevation [46]. The decreased activation in this region by religious and repetitive (Poem) conditions, makes us suggest that this interaction may be associated with the socio emotional regulation´s role of the mPFC. This effect, deactivation on mPFC during prayers, may explain the simple contrast between improvised conditions (Santa > Mother) where we observed a larger superior medial frontal cortex activation, partially overlapping with the mPFC interaction.

A previous study on Christian participants listening to intercessory prayers from speakers who they believed had superior hierarchy within their belief group showed similar deactivations in prefrontal and anterior cingulate regions when compared to less credible speakers [48]. This mPFC region has also been shown to exhibit reduced activation during emotional social processes [49]. Considering prayers are used in SYM to facilitate the state of mental silence, where practitioners experiment a spiritual union with God, we could associate the mPFC finding with the participants' attitude of surrendering towards God, in tune with a decrease of social judgment and ‘mentalizing’ functions reported in this region. Additionally, when the analysis was correlated with the years of the meditation experience, these regions showed larger deactivation. It would thus be interesting to test this effect in a more heterogenous study that included a wider range of experience, e.g., between novice and experienced practitioners of SYM.

In the main effect of secular speech, the deactivation of the thalamus during both prayers compared to secular conditions, could be explained by the role the thalamus in the conscious perception of the surroundings, controlling the flow of sensory information to the cortex [50]. Therefore, this deactivation could be linked to the inner concentration established during the prayers in order to reach a meditative state, reinforcing the downgrading of external distractors. Our previous functional connectivity study in SYM found a reduction in functional connectivity between the thalamus and the mPFC during the meditation state that was related to the depth of mental silence perceived by the subjects [38]. The thalamus has furthermore been shown to be reduced in activity in previous studies of different meditation techniques (yoga, transcendental meditation and mindfulness meditation), which associated the thalamic deactivation with reduction of pain perception, associating it with a filtering effect inflecting the sensory information [51–53]. This reduced thalamic activity was also found when comparing formalised secular speech with formalised prayer (Poem > Father), and perhaps could suggest a deeper focus during formal praying, but the effect did not appear in the equivalent improvised comparison (Santa > Mother) or in the contrast between the two kinds of prayers (Mother vs. Father).

**Fig. 3:**
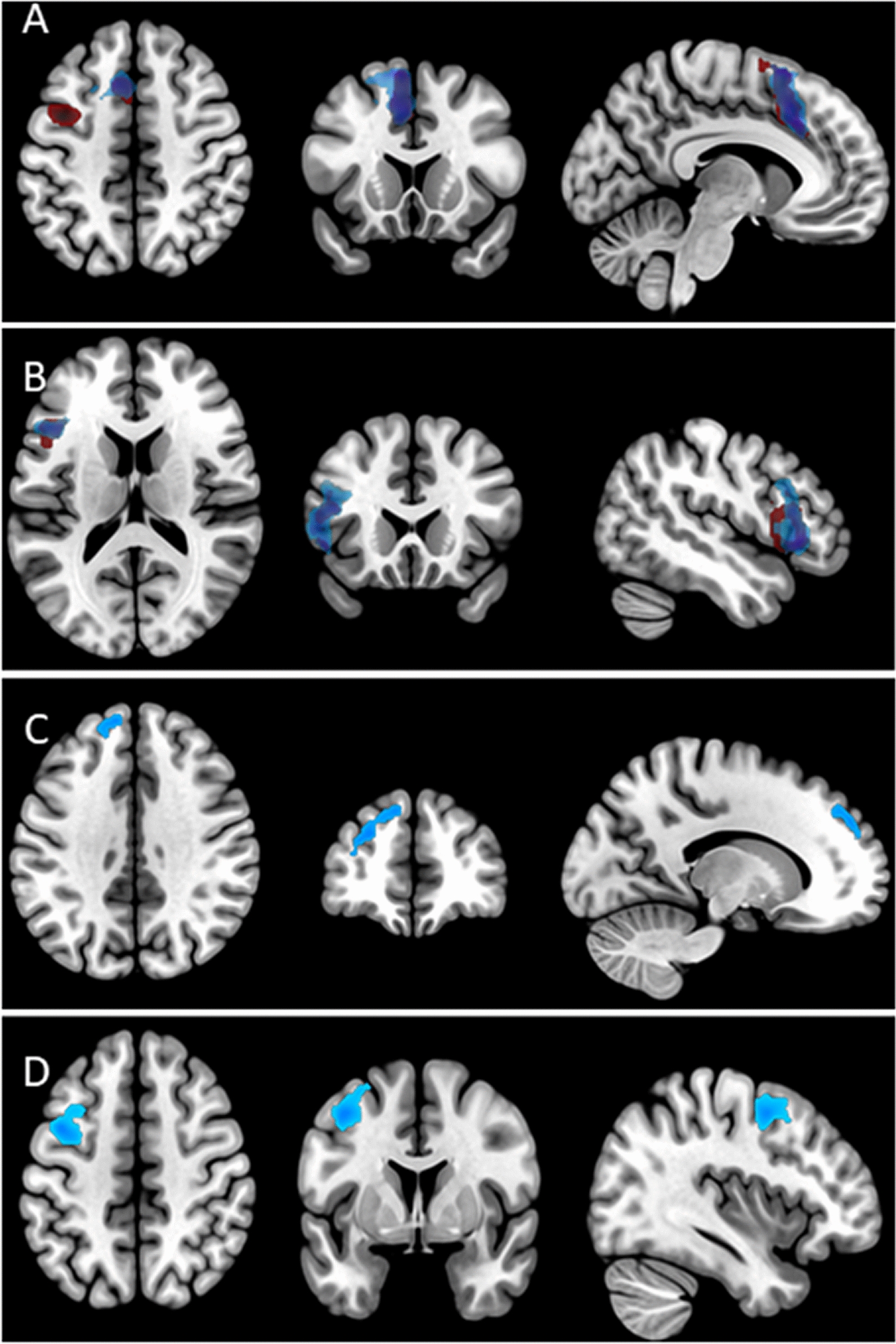
Main effects of improvised speech (Santa&Mother > Poem&Father) (blue), Mother > Father (red) and their overlaps (purple). **A** SMA; **B** vlPFC; **C** dlPFC; **D** Frontal eye Field/Pre-motor cortex. *SMA* supplementary motor area, *vlPFC* ventrolateral prefrontal cortex, *dlPFC* dorsolateral prefrontal cortex

In our study, the thalamus deactivation appeared to be the only cluster that separated the religious and secular speech conditions, since activations in the vlPFC, dlPFC and SMA were also observed in the contrast of improvised versus formalised speech (Santa & Mother > Poem & Father); some of these activation clusters also appeared in the simple contrast between improvised prayer versus formalised prayer (see Fig. 3), suggesting that improvised speech involved the activation of the left inferior frontal cortex (i.e., the Broca area), as well as the left SMA/dACC [54] and left dlPFC [55–57] in line with the known involvement of left hemisphere areas in language processes [58]. Thus, we observed that improvised speech involved left perisylvian brain regions, like the vlPFC which may be related to the social brain as well as language processes. Some of these regions also appeared in the simple contrast between formalised conditions, where the secular condition presented larger activation of the SMA and left dlPFC, which could be interpreted as a lower formal (automatic) effect of the poem in meditators, who pray frequently while the poem was, in some cases, particularly trained for the experiment.

In line with Schjoedt previous findings in Christian prayers [8], we also found no activation during prayers compared to secular recitations, which was also found in the individual contrasts comparing each prayer with its corresponding secular equivalent. The opposite contrast (Santa & Poem > Mother & Father) recruited the left dlPFC in both studies but also revealed several differences; while we obtained left hemispheric activation in the vlPFC, SMA, and thalamus, the original study from Schjoedt et al. showed right frontal activations alongside bilateral parieto-occipital activity, associating the bilateral frontal activity to an effect on the executive and attentional networks related to the participants’ different levels of familiarity with the two domains [8].

**Fig. 4:**
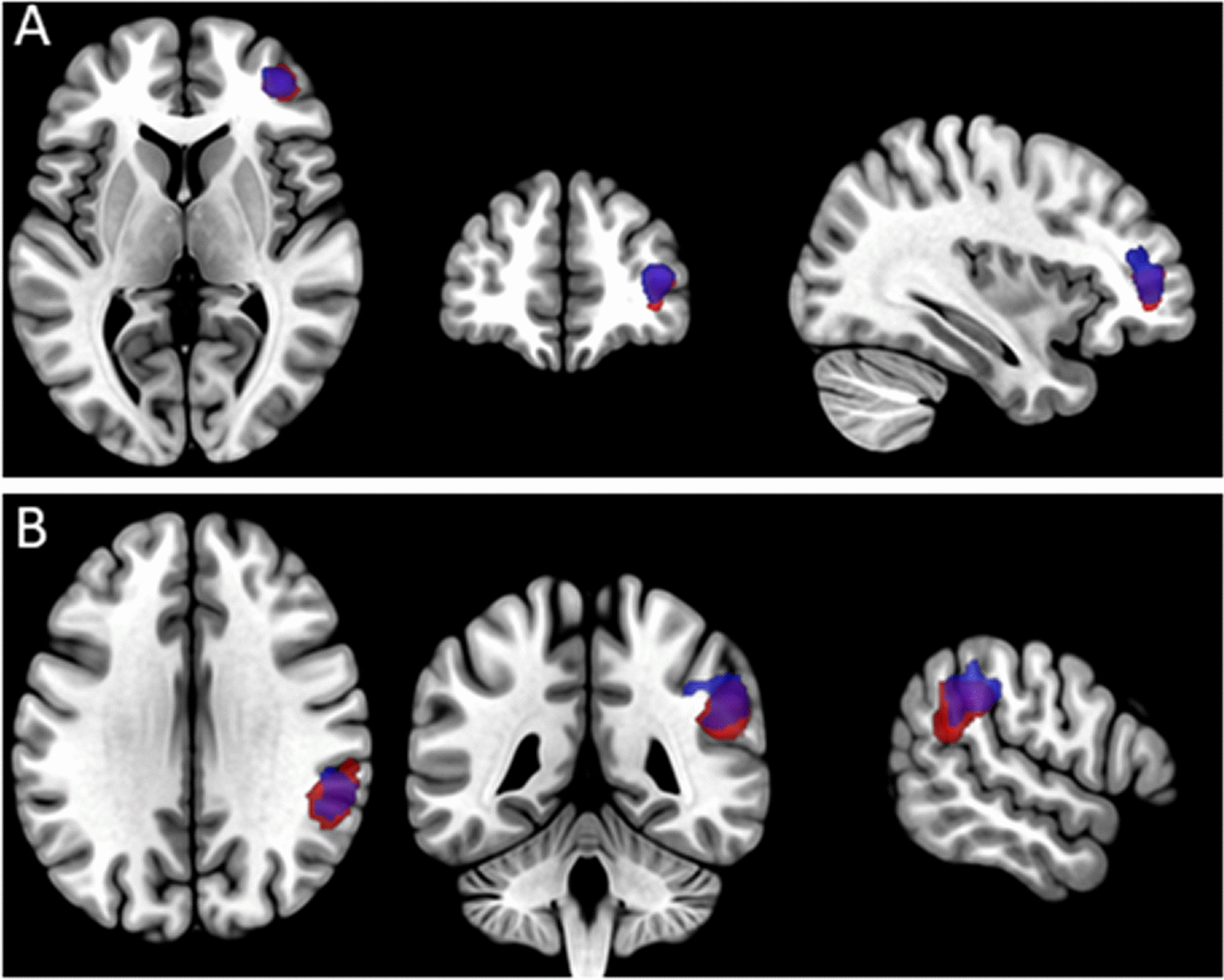
Main effects of formalised speech (Poem&Father > Santa&Mother) (blue), Father > Mother (red) and their overlaps (purple). **A** dlPFC; **B** IPC. *dlPFC* dorsolateral prefrontal cortex, *IPC* inferior parietal cortex

We also observed activations in the right dlPFC and right IPC associated with the formalised speech acts (Poem & Father) in SYM (see Fig. 4). This effect was also observed when comparing formalised prayer with both improvised conditions, but it cannot be uniquely associated with formalised prayer since it was not shown when comparing between the formalised speech acts. Similar regions were previously observed and associated with a general effect of rehearsal and retrieval [8], which further shows the aforementioned difference of familiarity between the formalised tasks. Therefore, we may suggest that formalised speech involves right lateralized brain regions such as the IPC and the dlPFC while improvised speech involved left hemispheric activations in vlPFC, dlPFC and SMA (see Figs. 3 and 4). Future studies exploring lateralization effects of formal prayer in social and language related brain regions may be of interest under the support of a strong hypothesis that is absent at this moment as far as we know.

## Limitations

The current study has several limitations. One limitation is the small sample size of volunteers participating in this study due to the low number of expert practitioners of SYM available in Tenerife. The second limitation concerns the absence of a resting task condition, as we used counting backwards as a baseline condition, mainly because an important aim was to replicate the study of Schjoedt [8]. However, this contrast with a mentally very demanding task could have diminished activation findings for the prayer and speech conditions. Additionally, some participants particularly trained the poem for this experiment, which may have negatively affected the results since participants have more practice with the formalised prayer. Finally, the quality of cognitive performance during the fMRI task could not be tested inside the scanner given the silent language conditions involved, which did not include any other behavioural record. We tried to control for task performance during fMRI acquisition using a self-report questionnaire after the scan session to reduce this limitation.

## Conclusions

In conclusion, this task-based functional magnetic resonance imaging (fMRI) research of the devotional-prayer aspect of SYM showed reduced frontal activity, particularly a deactivation in the mPFC during prayer, which may be related to the latter state of mental silence where participants perceive the state of yoga, which as previously mentioned is considered the spiritual union with God, showing their trust in the receiver of their speech (i.e., God or the founder of SYM).

We found several brain areas associated with social cognition to be involved in praying in SYM, similar to the ones presented in our study of reference, the study of Christian prayer [8]; however, the pattern of activation was different which could suggest that SYM prayer cognition differs from Christian prayer cognition. Different forms of praying appear to have some common elements but also reveal distinct cognitive elements and hence may have different regions of activation depending on the religious content and speech act. It would therefore be interesting to directly compare prayers in two or more different religious traditions.

Finally, the deactivation of the bilateral thalamus and frontal areas during both prayers compared to secular conditions, could be the main characterization of prayers in SYM, which could be explained by the role of the thalamus for the conscious perception of the surroundings, which is not necessary during praying and hence deactivated, and the surrendering attitude towards God, reflected in the deactivation of prefrontal regions. Therefore, these deactivations could be linked to the inner concentration and devotion during prayers that is part of the meditative process in SYM.

## Materials and methods

### Participants

Sixteen expert practitioners of Sahaja Yoga meditation volunteered to participate in this study (10 females; 1 left-handed; age range 24–63 years). All participants had more than 7 years of daily practice experience in this meditation. Volunteers reported (in a 5-point Likert scale) their experience in SYM, including among other things their frequency of praying the Lord’s Prayer and prayers to the Divine Mother, the average time dedicated to meditation per day and the frequency of the perception of the state of mental silence. After the scan session, participants were asked to assess the quality of their performance inside the scanner, and they had to complete a questionnaire on religious beliefs and practices, including belief in God, confidence in God’s reciprocity and frequency of praying (see Table 5). None of the participants reported any physical or mental illness, nor any history of neurological disorders, addiction to alcohol, nicotine or drugs.

**Table 5:** Demographic data and meditative and religious characteristics of the group

	Mean (SD)	Range
Age (years)	48.2 (10.3)	24.67 – 63.25
Education degree, 0 to 6	2.4 (1.6)	1 – 6
Years dedicated to SYM	19.6 (8.1)	7.0 – 30.0
Height (cm)	168.6 (7.7)	159.00 – 183.00
Weight (kg)	73.8 (12.8)	54.00 – 109.00
Minutes dedicated to meditation per day	47.1 (25.0)	20.00 – 120.00
Frequency of mental silence perception (1 to 5)	3.6 (1.1)	1 – 5
Lord’s Prayer weekly frequency	4.5 (3.8)	0 – 14
Mother Prayer weekly frequency	5.7 (3.6)	3 – 17
Do you believe in God? (1 to 5)	5 (0)	
Do you believe that Santa Claus exists? (1 to 5)	2.1 (1.1)	1 – 4
Confidence in God’s reciprocity? (1 to 5)	4.8 (0.5)	3 – 5

### Conditions and procedure

We designed a stimulus paradigm with five conditions based on Schjoedt’s previous neuroimaging study on prayer in Christian practitioners who belonged to a fraction within the Danish Lutheran Church called the Inner Mission [8, 17]. Four of the conditions followed a two-by-two factorial design between ‘Domain’ (spiritual vs. secular) and ‘Speech-act’ (formalised vs. improvised) factors (levels) (See Table 6). For the formalised spiritual speech-act, we used the recitation of the Lord’s Prayer (Father), and for the improvised spiritual speech-act, we used a prayer to the Divine Mother (Mother). For the secular speech acts, we used the recitation of a formalised well-known poem (Poem) and making improvised wishes to Santa Claus (Santa). We also included a control condition, similar to Schjoedt’s paradigm [8, 17], in which participants were asked to count backwards from 100 rather than a resting state baseline condition to avoid task versus resting related effects.

**Table 6:** Two-by-two factorial design

Two-by-two factorial design	Domain	
Speech Act	Religious	Secular
Formalised	Lord’s Prayer	Poem
Improvised	Mother Prayer	Wishes to Santa

In SYM, the Lord’s Prayer is mainly used by meditators when they focus their attention at the 6th center (called in ancient yoga Agnya Chakra) theoretically located according to SYM at the optic chiasm although meditators focus their attention on their forehead. This 6th center is related to the quality of forgiveness and the state of thoughtless awareness or mental silence [59]. On the other hand, the prayer to the Divine Mother is related to the 4th center (called in ancient yoga Anahata Chakra) located at the cardiac plexus and meditators put their attention around their heart zone; according to SYM the qualities of this 4th center among others are love, compassion, feeling of security and fearlessness [59] (some examples of prayer to the Divine Mother, written by participants of this study, and a diagram with the location of the chakras and their qualities according to SYM are available as supplementary data. See Additional file 1).

A prior 7 min of structural scanning was used to instruct volunteers to enter into meditation in order to facilitate the adaptation to the MRI scanner and also to simulate a normal SYM prayer condition which are normally recited inside a meditation state. Afterwards volunteers were asked to perform the already mentioned five tasks in a pseudo-randomized order. Each task was preceded by their respective two seconds auditory instruction. Each of the five conditions was repeated six times, each of them lasted 26 s, excluding the 2 s audio instruction. The total time of fMRI acquisition was 14 min. During this time, tasks were performed silently as internal speech with eyes closed, and participants were asked to concentrate on the task. They were instructed to repeat the current task if they finished it before the end of the 26 s block.

### MRI acquisition

Axially oriented blood-oxygen-level-dependent (BOLD) functional images were obtained by a 3T Sigma HD MR scanner (GE Healthcare, Waukesha, WI, USA) using an echo-planar-imaging gradient-echo sequence and an 8-channel head coil with the following parameters: repetition time (TR) = 2000 ms, echo time (TE) = 21,6 ms, flip angle = 90°, matrix size = 64 × 64 pixels, 37 slices, 4 × 4 mm in plane resolution, slice thickness = 4 mm, interleaved acquisition. The head was stabilized with foam pads. The slices were aligned to the anterior commissure—posterior commissure line and covered the whole brain. Functional scanning was preceded by 20 s of dummy scans to ensure tissue steady-state magnetization. A total of 420 fMRI whole brain volumes were taken during each participant’s single run.

Prior to the fMRI acquisition, high-resolution sagittal oriented anatomical images were collected for anatomical reference, for this purpose a 3D fast spoiled-gradient-recalled pulse sequence was obtained with the following parameters: TR = 8.844 ms, TE = 1.752 ms, flip angle = 10°, matrix size = 256 × 256 pixels, slice thickness = 1 mm.

### Data preprocessing and analysis

The functional brain images were pre-processed and analysed using Statistical Parametric Mapping, SPM12 (Wellcome Trust Centre for Neuroimaging, London United Kingdom). A general linear model was implemented for each participant at the first level effects, and the results were computed as a group (second-level effects).

Pre-processing steps included: (1) within-subject registration and unwarping of time series, the realignment part of this routine realigns a time-series of images acquired from the same subject using a least squares approach and a 6 parameter (rigid body) spatial transformation; (2) co-registering individual structural images to the mean functional image of each subject; (3) co-registered images were segmented into grey matter, white matter, cerebrospinal fluid, bone, soft tissue and air/background; (4) spatial normalization of functional volumes by using the parameters extracted from the anatomical segmentation procedure in each subject and resampling voxel size to 2 × 2 × 2 mm; and (5) spatial smoothing with an 8-mm full-width-at-half-maximum Gaussian kernel, a high pass filter was used to remove low-frequency drifts in the fMRI BOLD signal. Only one subject presented extensive head movement (more than 3 mm/3º), but movement was properly corrected and, thus, we included the subject in the analyses. We also tested whether the exclusion of this participant affected the group statistics and that was not the case.

The first level analysis included 6 conditions: baseline condition (counting backwards), the audio instruction of each task (which was not used for posterior analysis), and the four conditions of interest: Santa, Poem, Father and Mother. The contrasts of interest computed were as follows: (1) Domain main effect (Mother&Father > Santa&Poem and vice versa); (2) Speech act main effect (Poem&Father > Santa&Mother and vice versa). Next, we tested for interactions (Mother&Poem > Father&Santa and vice versa), simple effects (comparing Mother, Father, Santa and Poem with each other), and simple contrasts comparing each condition with the base-line condition (Count). Additionally, this analysis included the head motion parameters as regressors of no interest in the model. Group analysis was performed by using the random-effect approach with a one-sample t test.

We also explored the brain–behaviour relationships by a regression analysis with the frequency of practice (frequency of the Lord’s Prayer recitation, frequency of the Mother’s prayer and the frequency of mental silence perception) and the meditation experience as independent variables and brain activation as dependent variable. These analyses did not present extensive changes compared to the presented results and any relevant effect is discussed. Analyses were thresholded at a statistical voxel-wise *p* < 0.001 and at corrected cluster p-value with multiple correction *p* < 0.05 FWE.

## Supplementary Information

**Additional file 1: **Examples of spontaneous mother’s prayer, written by participants of this study.

## Data Availability

The datasets used and/or analysed during the current study are available from the corresponding author on reasonable request.

## Abbreviations

BOLD: Blood-oxygen-level-dependent
dlPFC: Dorsolateral prefrontal cortex
dmPFC: Dorsomedial prefrontal cortex
dACC: Dorsal anterior cingulate cortex
fMRI: Functional magnetic resonance imaging
MRI: Magnetic resonance imaging
mPFC: Medial prefrontal cortex
SMA: Supplementary motor area
SYM: Sahaja Yoga Meditation
TE: Echo time
TP: Temporal pole
TPJ: Temporoparietal junction
TR: Repetition time
vlPFC: Ventrolateral prefrontal cortex
