# Supplementary material for: Monitoring the neural activity associated with praying in Sahaja Yoga meditation
Source: BMC Neurosci. 2023 Nov 13;24:61. doi: 10.1186/s12868-023-00828-x (PMC10642040; doi:10.1186/s12868-023-00828-x)

**Additional file 1: Examples of spontaneous mother’s prayer, written by participants of this study.**

Mother, please come in my heart. Let me clean my heart so that You are there.

Mother Please open my heart so I can love the whole world and I can love myself.

Mother Please open my heart so I can have compassion for all human beings and I can also have compassion for myself.

Let me not be in delusion. Take me away from illusions. Keep me in reality. Take away the sheen of superficiality.

Mother please let feel the love of the Divine in my heart so I can share this love with everyone.

Mother please allow me to perceive and enjoy my spirituality please help me to connect with my true self the spirit

**Diagram with the location of the chakras and their qualities according to SYM.**


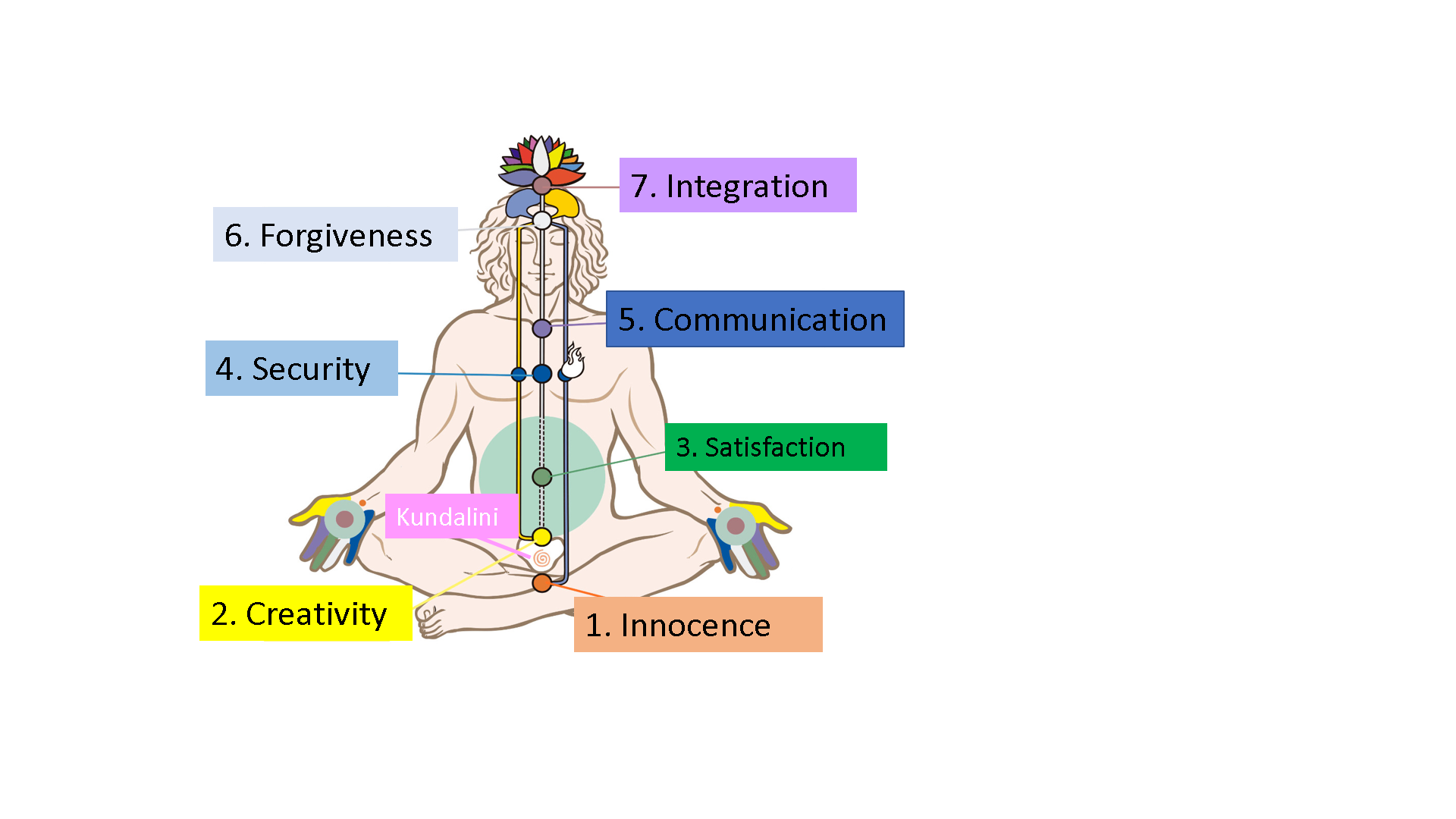

Supplement: Supplementary file 1 — Additional file 1: Examples of spontaneous mother’s prayer, written by participants of this study. [file 12868_2023_828_MOESM1_ESM.docx]
